# Fiscal stress as a catalyst for public service provision in China: evidence from the VAT reform

**DOI:** 10.3389/fsoc.2025.1610852

**Published:** 2025-09-10

**Authors:** Xiaoshan Cai, Bin Zhang, Jin Yang

**Affiliations:** ^1^School of Culture Tourism, Guangdong University of Finance & Economics, Guangzhou, China; ^2^Guangzhou Science and Technology Project Evaluation Center, Guangzhou, China; ^3^School of Management, Guangzhou College of Commerce, Guangzhou, China

**Keywords:** fiscal stress, public services provision, local government, China's VAT reform, generalized DID

## Abstract

Local government responses to fiscal stress are crucial for ensuring public service provision, especially in times of fiscal tightening. Previous studies have shown inconsistent findings, often focusing on developed countries in Europe and the United States. This study uses China's 2016 VAT reform as a quasi-natural experiment and applies a generalized difference-in-differences (DID) model to investigate the impact of fiscal stress on local government public service provision. The results reveal that fiscal stress promotes, rather than constrains, public service provision. The findings remain robust after a series of sensitivity tests. Furthermore, fiscal stress is found to be more conducive to enhancing public service provision in regions with lower fiscal decentralization, high-intensity intergovernmental competition, and higher fiscal transparency. This paper highlights the importance of a country's unique fiscal decentralization system and government performance evaluation goals in shaping local government behavior under fiscal stress. The findings provide valuable insights for improving fiscal allocation efficiency and guiding local government responses, with broader implications for similar studies in other developing countries.

## 1 Introduction

The emergence of global public health events such as the COVID-19 epidemic has further highlighted the importance of public services. It has become the consensus of governments around the world to improve provision of public services to meet the growing demand. The Chinese government has also put forward a series of plans for the provision of public services, and aims to build a more fair, effective, and efficient public services system to meet the people's growing needs for a better life.

Local government is the main provider of public services. In general, local government needs fiscal funds as a guarantee to provide public services. However, in the context of the global economic recession, the growing fiscal stress on local governments has become the focus of international attention, and how to allocate public service resources in a period of fiscal stress has become an important decision for local governments. Therefore, how do local governments respond to fiscal stress? As early as the 1970's, the theory of cutback management identified the above-mentioned issues as one of the main research topics (Levine, 1978; Behn, 1985; Bozeman and Straussman, 1982). Subsequently, the global financial crisis and the Great Depression continued to trigger attention to this issue. Previous literature has attempted to answer this question through case studies of the United States, as well as European countries such as the United Kingdom, Denmark, and Spain, but the conclusions drawn are not entirely consistent. China's fiscal system and government governance model are distinct from those of highly centralized European countries such as the UK, Portugal, Italy, Ireland, Denmark, and Spain. In these countries, the central government, under austerity conditions, often shifts stress and risks to local governments by cutting local spending, especially in public services. Local governments have little room to resist top-down austerity measures. It is also different from the federalism in the United States, where local governments have more autonomy both fiscally and politically. They are responsible for essential public services and, in times of austerity, tend to consider the public service needs of residents and use other tools to balance stress, often with a view to “voting with their feet.” In contrast, China implements a combination of vertical political management system with economic decentralization, with the central government setting administrative responsibility indicators and local governments responsible for implementation. In addition, since the implementation of the tax sharing reform in 1994, China's fiscal decentralization system has shown a trend of upward shift of fiscal power and decentralization of administrative power (Wang et al., 2024), most of the financial power belongs to the central government, and the expenditure responsibility of local governments has increased, sharpening the mismatch between the financial resources and expenditure responsibilities of the central and local governments. However, local governments in China also have a certain degree of discretion to use various methods to achieve fiscal balance. At the same time, China's local government has more robust public characteristics, it is a citizen-oriented government and creates public value through public administration. Furthermore, the performance targets of local governments in China are also changing, shifting from GDP to a multi-dimensional target system covering people's livelihood (Zuo, 2015). Based on these above characteristics and changes, the previous research perspectives and conclusions on developed Western countries may not necessarily be suitable for China's reality.

Based on this, this article extends the research samples from developed countries to developing countries. It takes China's VAT reform in 2016 as a quasi-natural experiment to discuss local governments' public services provision behavior under fiscal stress. In 2016, China implemented reform of replaced business tax with value-added tax and cuts tax and fee across the board, and China's macro tax burden has decreased yearly since 2016. The macro tax burden of 2015 to 2021 is 18.1%, 17.5%, 17.3%, 17.0%, 15.9%, 15.9%, 15.2%, and 13.2% respectively. Reducing the tax burden means the increase of government fiscal stress. From 2015 to 2021, the local fiscal self-sufficiency ratios^1^ were 55.21%, 54.41%, 52.80%, 52.02%, 49.61%, 47.56%, and 52.74%, respectively. It can be seen that since 2016, the local fiscal self-sufficiency rate has generally been on a downward trend, posing a severe challenge to the sustainability of local finances. Meanwhile, China's VAT reform is the top-level design of the national policy, which is not affected by local government behavior and fiscal conditions, and can alleviate endogeneity problems. So, it is reasonable to take China's VAT reform as a quasi-natural experiment. Based on this, this paper aims to explore how fiscal stress triggered by exogenous tax system reforms affects the public service expenditure behavior of local governments in China.

Compared with previous research, the potential contributions of this article are as follows: First, existing studies on government expenditure behavior under fiscal stress predominantly remain at the theoretical level of argumentation. This article uses the generalized DID model to analyze local governments' public services expenditure behavior under fiscal stress. It also discusses its heterogeneity under fiscal decentralization, intergovernmental competition, and fiscal transparency, verifies the existing theoretical view from the empirical perspective, and makes the research conclusion more convincing. Secondly, this article takes China's VAT reform in 2016 as a quasi-natural experiment of external “fiscal stress” impact. As a policy continuously implemented in China in the past decade, the scope and intensity of tax reduction in VAT reform are unprecedented. It is reasonable to use the tax reduction brought by the VAT reform as a representation of fiscal stress, and it can also observe local government behavior under fiscal stress over a longer period of time. Thirdly, local government expenditure behavior is influenced by fiscal decentralization. Traditional fiscal federalism posits that decentralization enhances the efficiency of local governments in providing public services. However, fiscal federalism treats the decentralization arrangement between central and local governments as an exogenous precondition and fails to consider the constraints and influence of the political system on a country's fiscal decentralization. Therefore, fiscal federalism lacks explanatory power for the expenditure behavior of local governments in developing countries, such as China. This article incorporates the national political institutional foundations of fiscal decentralization. By leveraging the unique institutional environment offered by China as a developing country, this article examines the impact of fiscal stress on local governments' public service provision. This approach extends the study of government behavior under fiscal stress to the context of emerging economies and developing countries, thereby enhancing the applicability and generalizability of the findings.

The rest of this article is arranged as follows: the second part is the literature review; the third part is theory and hypothesis presentation; the fourth part is methodology and data; the fifth part is the empirical results; the sixth part is the heterogeneity analysis; the seventh part is the discussions; the eighth part is limitations and future research directions; the ninth part is the conclusions and policy implications.

## 2 Literature review

How do local governments respond to fiscal stress? Some studies have shown that fiscal stress may distort the structure of public spending, leading local governments to cut public services and resulting in fluctuations in the quality of public services (Nelson and Balu, 2014; Levine, 1978; Zabalza, 2021). This view is called austerity urbanism, which holds that local governments often respond to fiscal stress by reducing services, privatizing and increasing royalties, leading to the reduction of public services quality and the loss of local public power, and forming a vicious circle (Kim and Warner, 2021; Peck, 2012). This theory has been tested in the UK, where local governments have low fiscal autonomy and about 60% of revenues come from the central government. This highly centralized fiscal system has led to the central government using limited fiscal funds for more critical spending during the Great Recession, and local governments have to significantly cut public services (Chapain and Renney, 2012; Meegan et al., 2014).

In contrast to the pessimistic view of austerity urbanism, pragmatic municipalism offers an optimistic view. The theory holds that austerity urbanism applies only to European countries with high central fiscal concentration and a small number of American cities. Kim and Warner (20160) and Warner et al. (2021) studied most American cities and found that local governments do not blindly cut public services when under fiscal stress. In contrast, they maintain public services by opening up new channels for increasing fiscal revenue, and innovating alternative public services, etc.

The theories of austerity urbanism and pragmatic municipalism describe two different behavioral paths of local government responses to fiscal stress. The reason is due to the institutional differences between countries and the performance assessment target. For example, in terms of the system, the United States is a federal country with high decentralization and a high degree of local autonomy. Under fiscal stress, local governments can implement alternative services and adopt alternative revenue tools. This autonomy is more conducive to local governments adopting balanced responses and implementing pragmatic municipalism. In contrast, the UK has a highly centralized fiscal management system. Fiscal of local governments mainly depends on the central government's fiscal allocations. When facing fiscal stress, the central government may adopt austerity fiscal policies, and limited fiscal autonomy of local government may result in a reduction in public services. Regarding government performance targets, local governments in the United States have relatively independence. The satisfaction of local people has an excellent influence on the tenure of local government officials. Based on the principle of “voting with their feet,” local governments will pay more attention to the needs of local people in public spending decisions (Tiebout, 1956). However, the fiscal autonomy of local governments in the UK is relatively weak. The government performance target will be more concentrated on the areas considered necessary by the central government. The local governments will cooperate with central government, and the public provision of local government will be more subject to the central government's decisions. It can be seen that the public services provision of local governments under fiscal stress is complex and inconsistent.

Although theories such as austerity urbanism and pragmatic municipalism have systematically analyzed behavioral strategies adopted by local governments in response to fiscal stress, these Western theories have limitations when applied to China due to differences in state-local structures and levels of decentralization. China's fiscal management model differs significantly from both the high centralization seen in the UK and some EU countries and the extensive decentralization and local autonomy characteristic of the United States. Instead, China adopts a unique system of “political centralization combined with economic decentralization,” which bears characteristics typical of administrative decentralization. Within this framework, the central government must maintain political and fiscal control over local governments. It incentivizes and constrains local governments' behavior through politically-aligned goal setting, personnel appointments, and performance evaluations. This allows the central government to maintain its authority while coordinating national priorities, including balancing multiple political-economic objectives, addressing regional disparities, and fulfilling the functional requirements of different government levels. While the central government grants local governments certain taxation rights and expenditure responsibilities, permitting autonomy over budget size and structure, this autonomy operates within centrally-defined policy frameworks and fiscal systems. Consequently, local decision-making is constrained by central policy goals and political assessments. Therefore, when facing fiscal stress, China's local governments often cannot simply passively cut public service expenditures as described by austerity urbanism in response to top-down austerity stresss. Instead, constrained by the central government's “public welfare” objectives, they adopt active strategies to balance fiscal stress, exhibiting certain federalist characteristics (Blanchard and Shleifer, 2001). Although pragmatic municipalism emphasizes the public welfare orientation of local governments and offers stronger explanatory power for local governments' behavior, the discretionary power of Chinese local governments under its fiscal decentralization system is limited. They cannot freely utilize various fiscal and revenue tools to address fiscal stress without intervention or assistance from central government. Consequently, local governments' behavior under fiscal stress may exhibit more “top-down” characteristics. China's political centralization and its unique configuration of fiscal authority and functional responsibilities imbue local government behavior under fiscal stress with distinct complexity. Analyzing such behavior thus requires consideration of China's foundational political institutions.

In summary, although the existing literature extensively discusses local government public service provision behavior under fiscal stress, the following research gaps persist. First, most studies focus on local governments' behavior in Western developed countries, particularly the United States and the United Kingdom, while lacking empirical analysis of developing countries, especially those with “hybrid regimes” like China. Second, prevailing theoretical frameworks (such as austerity urbanism and pragmatic municipalism) are primarily grounded in models of either highly decentralized or highly centralized fiscal systems without considering China's unique fiscal system of “political centralization combined with economic decentralization.” Therefore, neither austerity urbanism nor pragmatic municipalism is fully applicable to the Chinese context. When operating under fiscal constraints, Chinese local governments operate neither purely at the will of the central government nor through complete local fiscal autonomy. Instead, they act proactively under the central government's leadership. This characteristic has not been fully explored in existing literature. Finally, most relevant studies focus on theoretical derivation or case studies, lacking systematic empirical research in the context of developing countries. This study aims to fill the aforementioned research gap by focusing on China, which has a unique fiscal system, and employing a quasi-natural experimental design to empirically examine the impact of fiscal stress on local governments' public service provision.

## 3 Theory and hypothesis presentation

According to the existing theories, local governments responses to fiscal stress may be “austerity urbanism” or “pragmatic municipalism.” Adopting an approach of “austerity urbanism” will lead to a reduction in the provision of public services, thereby affecting citizen wellbeing and social equity (Peck, 2012; Donald et al., 2014). In contrast, local governments that choose a “pragmatic municipalism” strategy will strive to maintain service provision under fiscal stress (Scorsone and Plerhoples, 2010). This article holds that local governments in China will implement “pragmatic municipalism” to ensure the provision of public services under fiscal stress.

First of all, from the perspective of the fiscal system and the relationship between the central and local governments, unlike the fiscal federalism in the United States, the central government in China strengthened its macro-adjustment capabilities and assumed a dominant position in public fiscal after China's tax-sharing reform in 1994. The central government will conduct centralized decision-making to coordinate resources better and balance multiple interests when under fiscal stress. However, this centralization of central finance is also different from the UK. Although fiscal to maintain public services of local government under fiscal stress are limited, the central government will not blindly cut public services because the current Chinese government takes “protect people's livelihood” as an essential task. Based on the public characteristics of the Chinese government, the central government will prioritize public services under fiscal stress. Due to a “top-down” stress-based system, the goals of the central government will affect the decision of local governments, so they will be more inclined to adopt a pragmatic municipalism strategy through more proactive alternatives to ensure the provision and quality of public services.

Secondly, from the perspective of public budget theory, according to the public budgeting framework proposed by Foged (2022), participants in the public budget include “priority setters” and “spending advocates” with relatively successful access to resources when the fiscal stress is low, and the influence of priority setters will rise due to the long-term nature and sustainability considerations of tissue survival when the fiscal stress are high. The fiscal stress level changes the power relationship between priority setters and spending advocates. In China, the central government as a priority setter has more influence than local governments as spending advocates, and the central government will maintain provision of public services to ensure the country's stability and gain the people's support. Accordingly, we draw the following research hypotheses:

H1: Local governments will promote the public services provision when faced with fiscal stress.

The traditional theory of fiscal decentralization holds that compared with the central government, local governments are closer to voters, and understand the needs and preferences of residents, and have the advantages and motivation to provide public services. Therefore, the fiscal decentralization system helps to motivate local governments to improve the efficiency of public services provision (Seabright, 1996; Persson et al., 2000; Hindriks and Lockwood, 2009). However, the premise of fiscal decentralization to stimulate local governments to provide public services is that local governments have more autonomy and fiscal power to match their responsibilities. Since the implementation of tax sharing reform in 1994, the fiscal distribution pattern of central and local governments has been adjusted, with fiscal power gradually concentrated, while administrative power and expenditure responsibility are steadily shifted to local governments. According to the previous analysis, the central government will play a leading role and make overall decisions in the case of fiscal stress. Therefore, the traditional theory of fiscal decentralization on the public services provision under fiscal stress cannot be fully applied to China. In areas with low fiscal decentralization, local governments have limited fiscal autonomy and usually rely on transfer payments or fiscal subsidies to maintain public services provision (Dreyer and Schmid, 2015). And the central government will play a leading role in increasing transfer payments or fiscal subsidies to ensure the public services provision of these areas. In a high degree of fiscal decentralization, the local government has greater fiscal autonomy, sufficient tax sources can effectively meet the needs of public services expenditure, which leads to a relatively smaller impact of fiscal stress on the provision of public services. Accordingly, we draw the following research hypotheses:

H2: The promotion effect of fiscal stress on public services provision of local government is more pronounced in areas with low fiscal decentralization

According to local government competition theory (Tiebout, 1956), inter-jurisdictional competition will encourage local governments to use fiscal resources more prudently. To enhance competitiveness, local governments improve their attraction of labor, capital and other mobile factors to satisfy residents and business needs (Breton, 1989). In recent years, the performance assessment of the Chinese government has gradually shifted from focusing on GDP to a multi-dimensional mechanism considering people's livelihood. The guarantee on public services has become essential in local government competition and official promotion (Caldeira, 2012). Local governments face not only the central government's “top-down” performance appraisal mechanism but also the “bottom-up” public opinion supervision mechanism from the people. Under this dual constraint mechanism of direct and indirect constraints, local governments aim to provide better public services for competition. According to this competition goal, local governments in regions with high-intensity competition tend to consider not only economic development but also public services to avoid a disadvantage compared with other areas (Li and Zhou, 2004). Accordingly, we draw the following research hypotheses:

H3: The promotion effect of fiscal stress on public services provision of local government is more pronounced in areas with high-intensity intergovernmental competition.

Fiscal transparency reflects the open degree of government fiscal revenue and expenditure, which allows citizens to supervise local governments and is also beneficial for the central government to supervise local governments. On one hand, based on the principal-agency theory, citizens entrust local governments as agents to provide public services by paying taxes and simultaneously supervise local governments. The improvement of fiscal transparency helps citizens better grasp the fiscal revenue and expenditure of local governments, promote local governments to be more prudent and efficient in the allocation and use of fiscal funds, and effectively avoid the “moral hazard” (Persson et al., 2000; Benito and Bastida, 2009). Under fiscal stress, high fiscal transparency is more conducive to local governments to optimize the fiscal expenditure structure, encourage local governments to use fiscal funds better, and ensure the provision of public services to meet people's demand (Alt and Lassen, 2006; Montes et al., 2019). On the other hand, fiscal transparency can also alleviate the information asymmetry between the central and local governments. Through improving local government fiscal transparency, the central government can conduct a more comprehensive and objective assessment of local government officials, understand the actual fiscal situation of local governments, and design the transfer payment system more scientifically and reasonably. High fiscal transparency is more conducive to the central government alleviating the fiscal stress on local governments and ensuring the provision of public services (De Simone et al., 2019). Accordingly, we draw the following research hypotheses:

H4: The promotion effect of fiscal stress on public services provision of local government is more pronounced in areas with higher fiscal transparency.

## 4 Methodology and data

### 4.1 Model setting

The purpose of this research is to evaluate the effect of fiscal stress on local government provision of public services. Since the implementation of China's VAT reform provides a “quasi-natural experiment” and an exogenous shock for the fiscal stress change. Quasi-natural experiments usually refer to the method of assessing the effect of a policy or intervention by comparing the changes between the experimental and control groups under certain specific conditions under natural conditions. The DID method usually divides the samples into the treatment group and the control group, and evaluates the effect of policy implementation by comparing the differences between the treatment group and the control group before and after the policy intervention. However, the DID method needs to strictly set treatment group and the control group, and it is very difficult for our research object to distinguish such groups because all the province are affected by such a policy. Hence, we adopt a generalized DID method to conduct our research. The generalized DID method is applied by Nunn and Qian (2011) for the first time, who estimated the contributions of potato production to population and urbanization. The advantage of such a method is that it does not need to strictly divide the treatment group and the control group and thus can investigate the impact of an exogenous policy. This study draws on Chen's (2017) empirical strategy and use the generalized DID method for regression analysis, and selects 300 observed values from 30 provinces from 2012 to 2021 to build a panel data regression model. The VAT reform fully rolled out in 2016 was a national tax system adjustment carried out by the central government to optimize the tax structure and eliminate repeated taxation. The decision-making motivation behind this reform mainly stemmed from the macro-level tax system optimization considerations at the national level, rather than being designed or initiated in response to specific changes in the public service supply level of local governments. Therefore, it is reasonable to regard this policy reform as a policy shock exogenous to the public service behavior of local governments, avoiding the reverse causality problem of “changes in local public services leading to the reform.” The generalized DID model was constructed as follows:


(1) $$\begin{array}{l} {\text{Level}}_{\text{it }}\;=\;{\text{β}}_{\text{0}}\;+\;{\text{β}}_{\text{1 }}{\text{Stress}}_{\text{i}}\;\times \;{\text{Post}}_{\text{t}}\;+\;{\text{β}}_{\text{2}}{\text{X}}_{\text{it}}\;+\;{\text{Region}}_{\text{i}}\\ \;+{\text{Year}}_{\text{t}}\;+\;{\text{ε}}_{\text{it}} \end{array}$$

Where Level_it_ stands for provision of public services, Stress_i_ stands for fiscal stress, Post_t_ is the time dummy variable, which is 0 before 2016 and 1 after 2016. X_it_ is a series of control variables, i denotes the province and t denotes the year, and region is the region-fixed effect, the region-fixed effect refers to the fixed effect of the provincial local government, and year_t_ is the year-fixed effect. The interaction term Stress_i_ × Post_t_ is the core explanatory variable that this study focuses on, its coefficient β_1_ aims to estimate the impact of fiscal stress on the provision of public services after the implementation of the VAT reform. A positive and significant β_1_ suggests that the fiscal stress has a positive impact on the provision of public services, while a negative and significant β_1_ indicates that the fiscal stress could not promote provision of public services.

### 4.2 Variables and data

#### 4.2.1 Dependent variable

The dependent variable of this study is provision of public services (Level). Based on China's 14th Five Year Plan for Public Services and National Basic Public Service Standards, this study selects the tier one indicators from three dimensions of sense of gain, happiness, and security. Further on, nine tier two indicators are selected including elderly care security, social security, employment security, education services, medical and health services, public cultural services, community services, infrastructure, environmental protection. Finally, we get 26 tier three indicators to establish the public services provision index system. Table 1 shows the indicator system of public services provision. The entropy method (Srdjevic et al., 2004; Han et al., 2022; Dai et al., 2023) was used to measure the level of public services provision in 30 provinces in China from 2012 to 2021. To conduct the robustness test, the Data Envelopment Analysis (DEA) method is adopted to measure the efficiency of public services provision (Effi) in 30 provinces in China from 2012 to 2021.

**Table 1:** Indicator system of public services provision.

**Tier one indicators**	**Tier two indicators**	**Tier three indicators**	**Indicator code**	**Unit**	**Weight**
Sense of gain	Elderly care security	Pension insurance participation rate for urban and rural residents	X1	%	1.51%
		Number of beds for the aged per thousand elderly population	X2	Sheet	1.64%
	Social security	Unemployment insurance participation rate	X3	%	5.75%
		Medical insurance participation rate	X4	%	2.74%
		Industrial injury insurance participation rate	X5	%	4.88%
		Maternity insurance participation rate	X6	%	4.62%
	Employment security	The number of people who receive career guides in this period	X7	Thousands of people	4.75%
		The number of people who receive entrepreneurial services in this period	X8	Thousands of people	6.74%
Sense of happiness	Education services	Kindergartens per capita	X9	Place	2.05%
		Primary schools per capita	X10	Place	3.00%
		Junior high schools per capita	X11	Place	1.60%
		Secondary vocational schools per capita	X12	Place	2.03%
	Medical and health services	Number of beds in medical and health institutions per 1,000 people	X13	Sheet	1.45%
		Number of health facilities per thousand people	X14	Individual	1.64%
		Number of health technicians per thousand people	X15	Person	1.76%
	Public cultural services	Performance sessions of art performance groups	X16	Ten thousand plays	9.39%
		Mass cultural institutions have held training courses	X17	Ten thousand times	4.80%
		Per capita public library collection	X18	Book	4.89%
		Number of public museum collections per capita	X19	Piece	5.14%
Sense of security	Community service	Number of community health service centers per 10,000 people	X20	Individual	4.51%
	Infrastructure	Total annual water provision for domestic water use	X21	Ten thousand cubic meters	5.02%
		Annual total natural gas provision	X22	Ten thousand cubic meters	5.27%
		Number of city road lights	X23	Thousand lanterns	4.71%
	Environmental protection	Per capita park green space area	X24	Square meter	1.30%
		The total number of special vehicles and equipment for urban sanitation per 10,000 people	X25	Table	4.42%
		Daily urban sewage treatment capacity	X26	Ten thousand cubic meters	4.39%

Data sources: China Statistical Yearbook, China Social Statistical Yearbook, China Labor Statistics Yearbook, China Regional Economic Statistics Yearbook, China City Statistical Yearbook, China Fiscal Transparency Report.

#### 4.2.2 Independent variable

The independent variable is fiscal stress (stress). As we take the implementation of China's VAT reform as a quasi-natural experiment, the fiscal stress raised from this reform should comprehensively consider the fiscal decline brought about by the cancellation of business tax, and the fiscal supplement brought about by the adjustment of value-added tax sharing ratio. This study draws on the method proposed by Chen (2017) to measure the fiscal stress faced by local governments by comparing the relative size differences between value-added tax and business tax before and after the reform. The specific measurement method is as follows:

Firstly we estimate the fiscal impact from value-added tax:


(2) $$\begin{array}{l} {\text{Stress}}_{\text{i,1}}=\frac{1}{4}\sum_{2012}^{2015}\frac{\left(\text{v}at_{i,t}+subsidy_{i,t}^{1}\right)}{revenue_{i,t}}\\ \;-\frac{1}{6}\sum_{2016}^{2021}\frac{\left(vat_{i,t}+subsidy_{i,t}^{1}\right)}{revenue_{i,t}} \end{array}$$

Secondly, we estimate the fiscal impact from business tax:


(3) $$\begin{array}{l} {\text{Stress}}_{\text{i},2}=\frac{1}{4}\overset{2015}{\underset{2012}{\sum }}\frac{\left(\text{s}aletax_{i,t}+subsidy_{i,t}^{2}\right)}{revenue_{i,t}}-\frac{1}{6}\overset{2021}{\underset{2016}{\sum }}\frac{subsidy_{i,t}^{2}}{revenue_{i,t}} \end{array}$$

Finally, we estimate the overall impact of VAT reform on the fiscal stress of local governments:


(4) $$\begin{array}{l} {\text{Stress}}_{\text{i}}={\text{Stress}}_{\text{i,1}}+{\text{Stress}}_{\text{i,2}} \end{array}$$

Combining Equations 2–4, we get Equation 5 as follows:


(5) $$\begin{array}{l} {\text{Stress}}_{\text{i}}\;={\text{Stress}}_{\text{i,1}}+{\text{Stress}}_{\text{i,2}}\\ \;=[\frac{1}{4}\sum_{2012}^{2015}\frac{\left(vat_{i,t}+subsidy_{i,t}^{1}\right)}{revenue_{i,t}}-\frac{1}{6}\sum_{2016}^{2021}\frac{\left(vat_{i,t}+subsidy_{i,t}^{1}\right)}{revenue_{i,t}}]\\ \;+[\frac{1}{4}\sum_{2012}^{2015}\frac{\left(saletax_{i,t}+subsidy_{i,t}^{2}\right)}{revenue_{i,t}}-\frac{1}{6}\sum_{2016}^{2021}\frac{subsidy_{i,t}^{2})}{revenue_{i,t}}]\\ \;=[\frac{1}{4}\sum_{2012}^{2015}\frac{\left(vat_{i,t}+saletax_{i,t}+subsidy_{i,t}\right)}{revenue_{i,t}}-\frac{1}{6}\sum_{2016}^{2021}\frac{\left(vati,t+subsidy_{i,t}\right)}{revenue_{i,t}}] \end{array}$$

Stress_i_ stands for the fiscal stress of local government, vat_i, t_, saletax_i, t_, subsidy_i, t_, revenue_i, t_ respectively refer to the value-added tax revenue, business tax revenue, other revenue (including tax refunds, superior subsidies and transfer payments, etc.) and general public budget revenue of local government. Assuming that local government is only affected by business tax and value-added tax, ${\text{subsidy}}_{\text{i},\text{t}}^{1}$ refers to the amount of other income of local government affected by value-added tax, ${\text{subsidy}}_{\text{i},\text{t}}^{2}$ refers to the amount of other local government revenue affected by business tax, and subsidy_i, t_ = ${\text{subsidy}}_{\text{i},\text{t}}^{1}$ + ${\text{subsidy}}_{\text{i},\text{t}}^{2}$. If stress_*i*_ > 0, it indicates that the fiscal resources of province i are weakened and the fiscal stress increases. Moreover, the larger the value, the greater the fiscal stress. Conversely, if stress_*i*_ ≤ 0, the fiscal stress is alleviated. In addition, the national fiscal statistics of cities and counties of other income (subsidy_i, t_) data have not been updated since 2009, so we cannot obtain the relevant data during the sample period. From the perspective of maintaining fiscal balance, this study assigns subsidy_i, t_ is equal to the general public budget expenditure minus the local government general public budget expenditure. Since the transfer payment is generally less negative, this study use the absolute value of local government general public budget expenditure minus general public budget revenue for subsidy_i, t_. While this proxy may introduce potential biases by incorporating non-tax-related fiscal imbalances or discretionary expenditure changes, it still serves as a useful estimate for capturing exogenous stress on local fiscal capacity caused by structural tax changes.

#### 4.2.3 Control variables

Control variables are selected in terms of regional characteristics including economic development level, industrial structure, government size, non-tax revenue, residents' education level, and population density. (1) Economic development is a sufficient condition to maintain the provision of public services. In areas with higher level of economic development, local governments have more fiscal resources to promote public services. So we choose economic development level (GDP) as control variable. (2) Industrial development has driven the transfer of population, bringing more labor and capital to the region, and playing a positive role in solving the employment stress and encouraging social forces to participate in the provision of public services. So we choose industrial structure (Struc) as control variable. (3) The relationship between government size and public services has a dual nature. On the one hand, as the size of the government expands, its capacity of public services provision also increases accordingly. On the other hand, if the government size is too large, it will lead to an increase in administrative expenses and will instead weaken the level of public services provision. So we choose government size (Size) as control variable. (4) Non-tax revenue is part of local government fiscal revenue and is also a source of funds for the provision of public services. So we choose non-tax revenue (Non-tax) as control variable. (5) The higher the residents' education level, the stronger the residents' ability to identify and supervise government actions, which is more conducive to promoting local governments to improve the level of public services provision. So we choose residents' education level (Edu) as control variable. (6) The higher the population density, the greater the demand for public resources such as education, medical and health care, and higher requirements for local government public services provision. So we choose population density (Density) as control variable. Table 2 shows the descriptions for each variable.

**Table 2:** Definition of major variables.

**Type**	**Variable**	**Abbr**.	**Definition**
Dependent variable	Public services provision	Level	Calculated based on the public services provision indicator system
		Effi	Calculated based on the input-output of public services. Input indicators: Six per capita fiscal expenditures on social security and employment, education, health care, culture, tourism, sports and media, urban and rural communities, and energy conservation and environmental protection Output indicators: public services provision
Independent variable	Fiscal stress	Stress	The relative size differences between value-added tax and business tax before and after the reform, calculated based on Equation 5
Control variable	Economic development	GDP	Logarithm of the per capita GDP of each province
	Industrial structure	Struc	Proportion of the added value of the tertiary industry to the region's GDP
	Government size	Size	Ratio of local general public budget expenditures to GDP
	Non-tax revenue	Non-tax	Proportion of non-tax revenue within the local government's general public budget
	Residents' education level	Edu	Proportion of high school and junior college or above among the population over 6 years old
	Population density	Density	Logarithm of the number of people per square kilometer

#### 4.2.4 Data specification and summary statistics

Our selected sample covers a dataset of province-level in China from 2012 to 2021. While provincial governments are critical players in public service provision, sub-provincial entities, such as municipalities and counties, often face different fiscal realities and are the primary actors in service delivery. However, due to data availability constraints, detailed fiscal data for sub-provincial levels is not readily available, making it difficult to analyze the behavior of local governments at lower administrative levels. Therefore, provincial-level data was chosen as the best available alternative to explore the impact of fiscal stress on public service provision. This limitation is discussed further in the section on the study's limitations and future research directions. Based on province-level data, 30 provinces^2^ in China from 2012 to 2021 were selected as the sample excluded Tibet, Hong Kong, Macao, and Taiwan which with extensive missing data. In addition, the period from 2012 to 2021 is chosen as the sample period because China's VAT reform was implemented in 2016, so there are window periods before and after the reform implemented. The data are obtained from the China Statistical Yearbook, China Social Statistical Yearbook, China Labor Statistics Yearbook, China Regional Economic Statistics Yearbook, China City Statistical Yearbook, China Fiscal Transparency Report, the China Municipal Government Fiscal Transparency Research Report, the Provincial Government Work Report, the Ministries Website, etc. Table 3 reports summary statistics of all the variables.

**Table 3:** Summary statistics.

**Variable**	***N***	**Mean**	**Sd**	**Min**	**Max**
Level	300	0.215	0.0730	0.0925	0.435
Effi	300	0.949	0.0892	0.546	1
Stress	300	−0.256	0.241	−1.030	0.0399
GDP	300	10.91	0.429	9.889	12.12
Struc	300	0.484	0.0945	0.309	0.839
Size	300	0.251	0.103	0.107	0.643
Non-tax	300	0.266	0.0741	0.0400	0.430
Edu	300	0.318	0.0929	0.122	0.699
Density	300	5.472	1.296	2.068	8.282

## 5 Empirical results

### 5.1 Baseline regression results

We used a generalized DID model to analyze the effects of fiscal stress on the provision of local government public services based on Equation 1. The results of the baseline regression are shown in Table 4. Columns (1) is the basic regression result without adding other control variables. It can be seen that the regression coefficient is 0.039 and significant at the 1% level, which suggests that fiscal stress can promote the provision of local government public services. Column (2) added control variables, and after controlling region and year fixed effect, the coefficient of stress is 0.034, which is also significant at 1% level. This indicates that for every standard deviation (0.241) increase in local fiscal stress, the provision of public services of local governments will increase by about 0.8%. The baseline regression results not only directly confirm the reverse pressure mechanism of fiscal stress on local government public services provision but also indirectly reflect the governance strategy of local governments to improve efficiency and reduce stress under China's “stress-based system.” This empirical results support hypothesis 1.

**Table 4:** Baseline regressions.

**Variable**	**Level**	**Level**
	**(1)**	**(2)**
Stress	0.039^***^	0.034^***^
	(0.009)	(0.009)
GDP		0.058^***^
		(0.017)
Struc		0.029
		(0.043)
Size		0.173^***^
		(0.056)
Nontax		−0.001
		(0.030)
Edu		−0.039
		(0.059)
Density		0.004
		(0.043)
Region FE	Yes	Yes
Year FE	Yes	Yes
Observations	300	300
*R*-squared	0.880	0.889

The values in parentheses are clustering with a robust standard error, ^*^, ^**^, and ^***^ represent the significance levels of 10%, 5%, and 1%, respectively.

In recent years, due to the multiple impacts of the COVID-19 epidemic and the international environmental situation, China has faced severe challenges regarding balance of payments. However, in the context of fiscal stress, the central government has actively responded with more excellent macroeconomic policies and pointed out that people's livelihood expenditures should occupy a priority position in the fiscal budget, and local governments also insisted on ensuring people's livelihood, wages and operations. It can be seen that in the face of fiscal stress, China's central government is more dominant, and publicity is also the internal essence of the Chinese government. Therefore, China's local governments practice “pragmatic municipalism” when faced with fiscal stress.

### 5.2 Parallel trend test

An essential requirement of the DID method is that the parallel trend assumption must be met. That is, before implementing of the VAT reform, the changing trend of public services provision was consistent in the regions affected by different fiscal stress. Use the significance level of the pre-event coefficients as the criterion for evaluating the parallel trends assumption, if the pre-event coefficients are not significant, then the parallel trends test is passed. This study draws on Beck et al. (2010) and employs the event study method to conduct a parallel trends test, and discusses the dynamic impact of VAT reform on public service provision. We expanded model (1) as follows:


(6) $$\begin{array}{l} {\text{Level}}_{\text{it}}=\alpha_{0}+\sum_{\text{t }=\;2012}^{\text{t }=\;2021}\alpha_{t}{\text{stress}}_{\text{i}}\times {\text{post}}_{\text{t}}+\alpha_{2}{\text{X}}_{\text{it}}+{\text{region}}_{\text{i}}+{\text{year}}_{\text{t}}\\ \;+\;{\text{ε}}_{\text{it}} \end{array}$$

**Figure 1:**
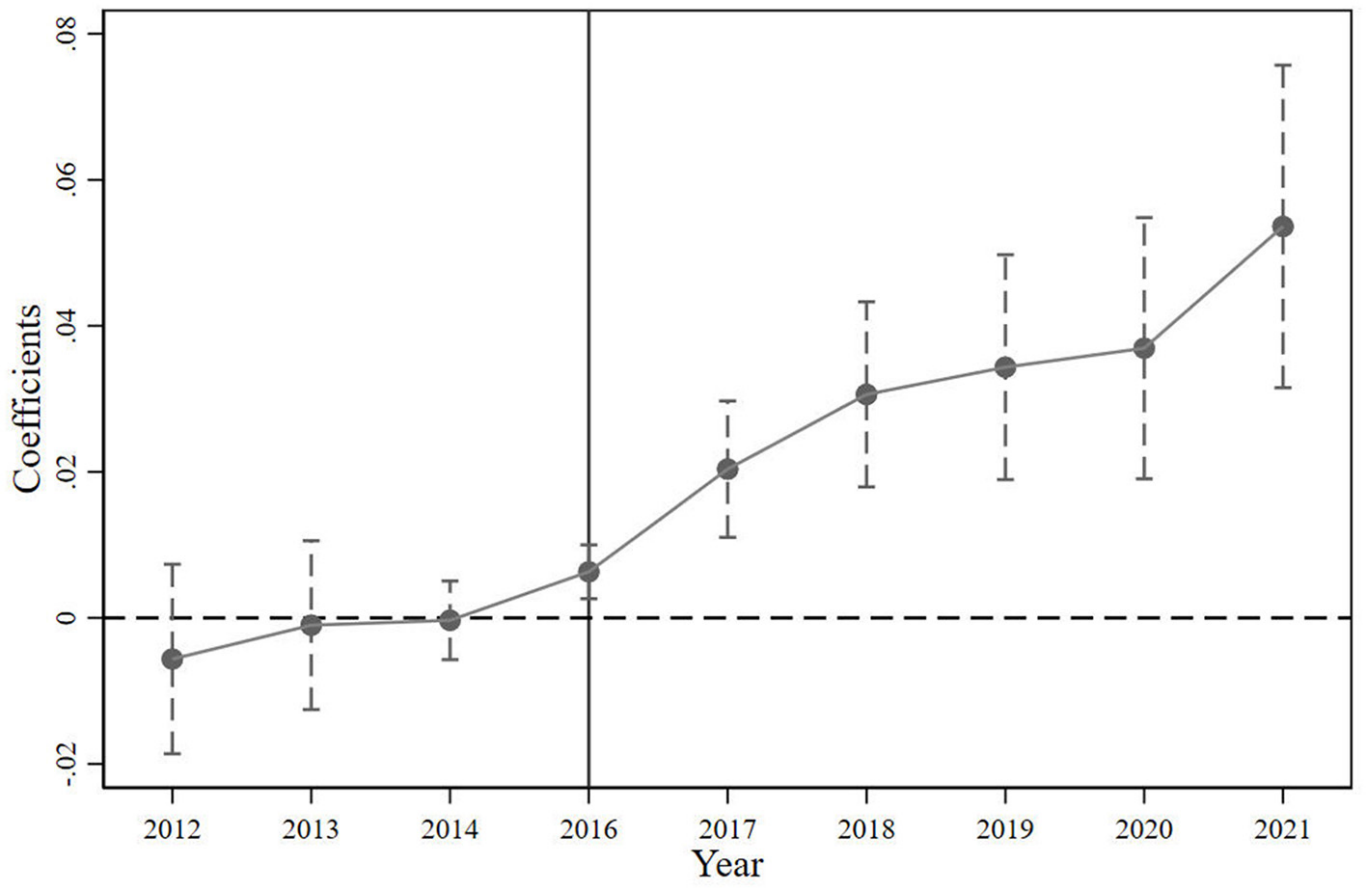
Parallel trend test.

In Equation 6, post_t_ is the dummy variable representing each year from 2012 to 2021. The value is 1 for the current year and 0 for other years. Other variables are defined similarly as in model (1). This study chooses the year before the reform (2015) as the benchmark group because the policy did not directly or indirectly impact the research indicators in the year before the policy was implemented. Using this period as the benchmark, the group can better observe changes after implementing the policy. The results of the parallel trend test are shown in Figure 1. The coefficient of the intersection term is not significantly before the implementation of the VAT reform (2016), indicating that the sample public services provision meets the parallel trend. The estimation result of generalized DID is effective. After the policy shock, the coefficient of the interaction term is significantly positive, indicating that fiscal stress brought about by the VAT reform has a significant promoting effect on public service provision, and this influence generally shows a gradually increasing trend. It shows that although the VAT reform has increased the fiscal stress on local governments, the central government will prioritize people's livelihoods as a guiding principle, prioritize public services as budget expenditures, and increase transfer payments to local governments. Local governments will also implement the central government's decisions to protect people's livelihood, effectively ensure and improve the provision of public services, and strengthen fundamental, inclusive, and bottom-up livelihood construction. In 2024, the Chinese government budget report indicate that local governments should coordinate the transfer payments from superiors and their own fiscal, prioritizing fiscal to guarantee people's livelihoods, wages, and operation (referred to as the “three guarantees”). Before the budget for “three guarantees” is fully allocated, no other expenditure budget shall be arranged. It can be seen that Chinese local governments have the determination and action power to ensure people's livelihoods when faced with fiscal stress.

### 5.3 Parallel trend sensitivity test

**Figure 2:**
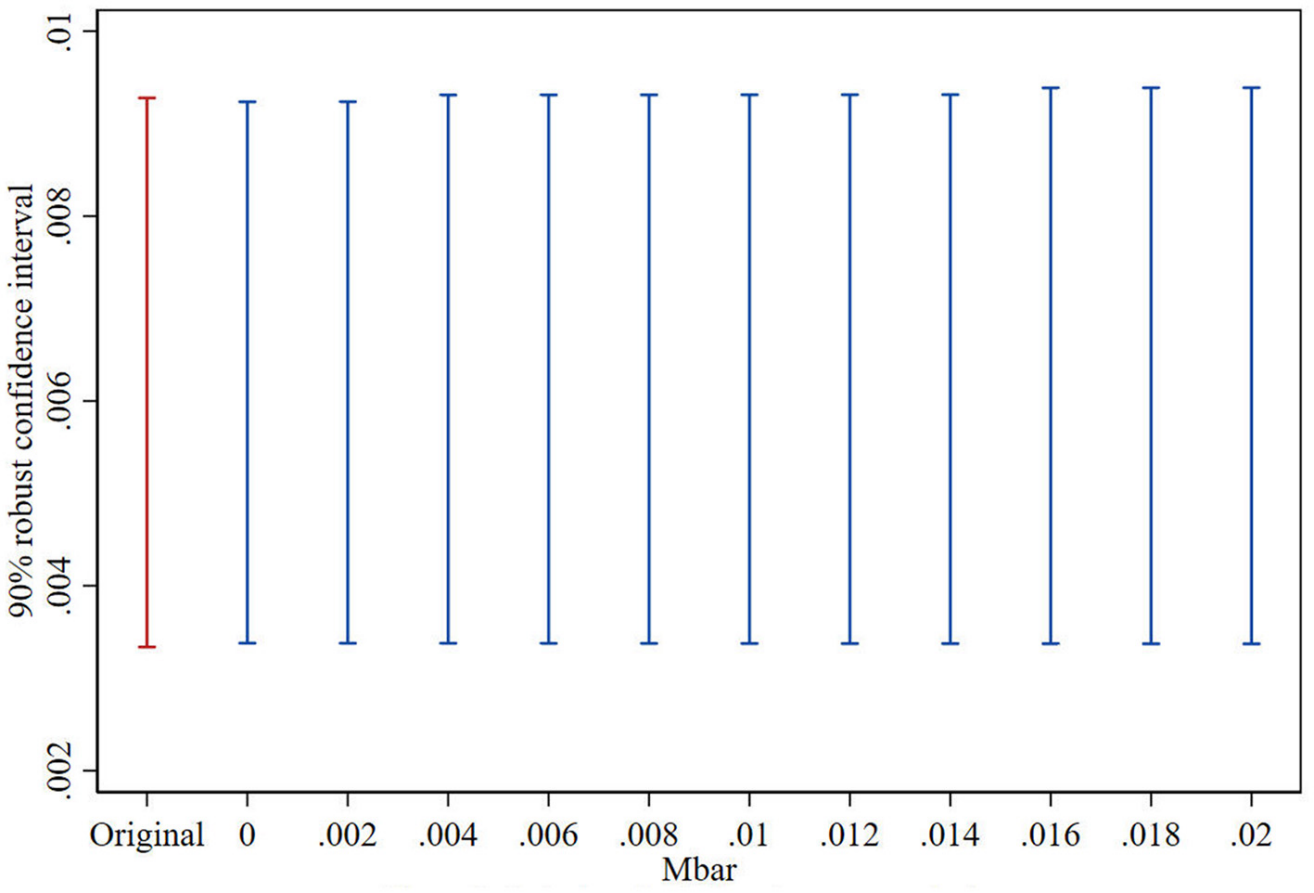
Relative deviation degree restrictions.

**Figure 3:**
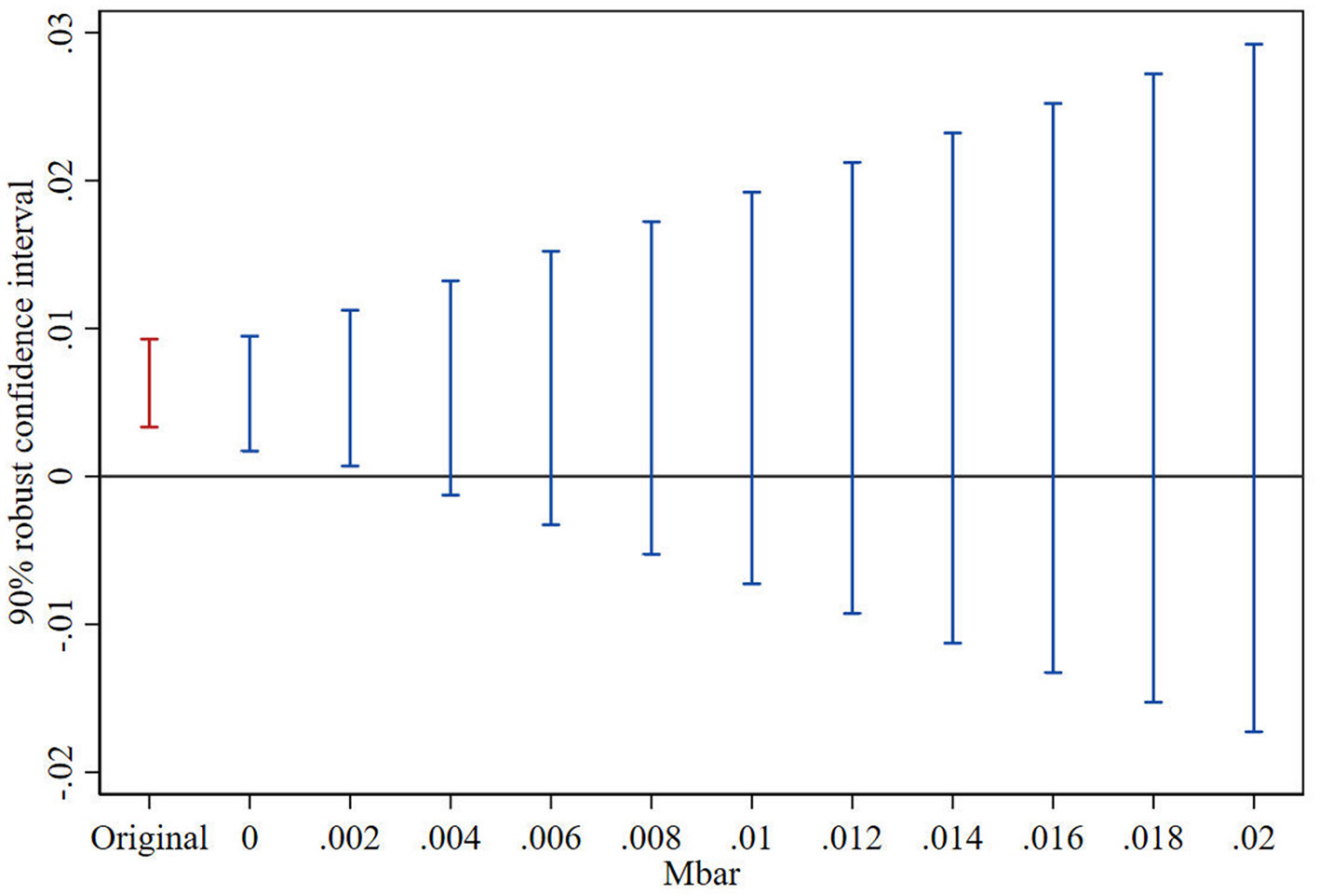
Smoothness restrictions.

This study adopts the Honest DID method to further explore the reliability of the parallel trends test results under the scenarios of relative deviation degree restrictions and smoothness restrictions (Rambachan and Roth, 2019; Clarke and Mühlrad, 2021; Biasi and Sarsons, 2022; Rambachan and Roth, 2023). The test consists of two parts: firstly, constructing the maximum deviation degree (Mbar) of the parallel trend; secondly, constructing the confidence interval of the post-treatment point estimator corresponding to the above deviation degree. If under the situation of the maximum deviation degree, the confidence interval of the post-treatment point estimator does not contain the value of 0, it indicates that the treatment effect has good robustness to the deviation degree of the parallel trend. This paper follows the approach of Biasi and Sarsons (2022) and sets the maximum deviation degree as Mbar = 1 × standard error, to examine the parallel trend sensitivity of the treatment effect after the VAT reform. Figures 2, 3, respectively, show the results of the parallel trend sensitivity tests for the treatment effects in the year of VAT reform implementation. Under the relative deviation degree restriction, the impact of fiscal stress on public service provision in the year of VAT reform implementation is very robust. Under the smoothness restriction, When M <0.002, the robust confidence set contains only positive values. That means when the pre-treatment trend deviation is less than 10%, the confidence interval of the treatment effect is still different from zero, the impact of the VAT reform on public service provision in the year of implementation is still robust. The test results show that even with some deviation in the parallel trend, China's VAT reform still has a significant positive impact on public services provision of local government.

### 5.4 Robustness test

The robustness test is conducted to ensure the reliability of the aforementioned results by replacing independent variable and dependent variable, replacing the sample interval and the placebo test.

First, we replace the fiscal stress variable with a dummy variable (Stress2), and re-estimated the model using a standard difference-in-differences (DID) specification with discretely defined grouping variables. Specifically, using the median fiscal stress (Stress) as the threshold, regions with fiscal stress above this value are defined as the treatment group (Stress2 = 1), while regions with fiscal stress equal to or below this value are defined as the control group (Stress2 = 0), so as to build 0–1 type treatment grouping variables and further use the traditional DID for regression. The regression results are shown in column (1) of Table 5. It shows that the coefficient of the Stress2 is significantly positive at the level 5%. Which verifies that the fiscal stress has a positive impact on the public services provision and the robustness of the research conclusion.

**Table 5:** Robustness test.

**Variable**	**(1)**	**(2)**	**(3)**
	**Level**	**Effi**	**Level**
Stress2	0.011^**^		
	(0.005)		
Stress		0.061^*^	0.033^***^
		(0.035)	(0.009)
Control variables	Yes	Yes	Yes
Region FE	Yes	Yes	Yes
Year FE	Yes	Yes	Yes
Observations	300	300	240
*R*-squared	0.885	0.248	0.873

The values in parentheses are clustering with a robust standard error, ^*^, ^**^, and ^***^ represent the significance levels of 10%, 5%, and 1%, respectively.

Second, we replace the independent variable with the efficiency of public services provision (Effi) to conduct a robustness test. Efficiency of public service provision reflects the efficiency of local government in providing public services, improving the efficiency of public services provision will also improve the public services provision. The regression results are showed in column (2) of Table 5. It shows that the fiscal stress's estimated coefficient is still significant at the 10% level. Specifically, for every one standard deviation (0.241) increase in local fiscal stress, the efficiency of local government public services provision will increase by about 1.5 percentage points, indicating that the conclusions obtained in this study are still robust.

Third, we replace the sample interval to exclude the impact of the COVID-19 pandemic on local governments' public service provision. The sample interval of this study is from 2012 to 2021. However, since the COVID-19 pandemic broke out in 2020, the pandemic may lead to changes in the public's demand for public services, such as an increased demand for epidemic prevention emergency supplies and online public services, the already strained fiscal resources were increasingly allocated to emergency management and public services in special times. Given the specificity of the pandemic could potentially cause bias into the research conclusions, this study excludes the data during the pandemic period and replaces the sample interval to 2012–2019. As shown in column (3) of Table 5, the regression result indicates that the estimated coefficient of fiscal stress remains significant at the 1% level. This result shows that the research conclusion has not changed significantly, further confirming the robustness of the findings. This test result also reflects that, even under the extreme circumstances of COVID-19 pandemic, local governments in China still make fiscal expenditure choices to ensure basic public services for the public.

**Figure 4:**
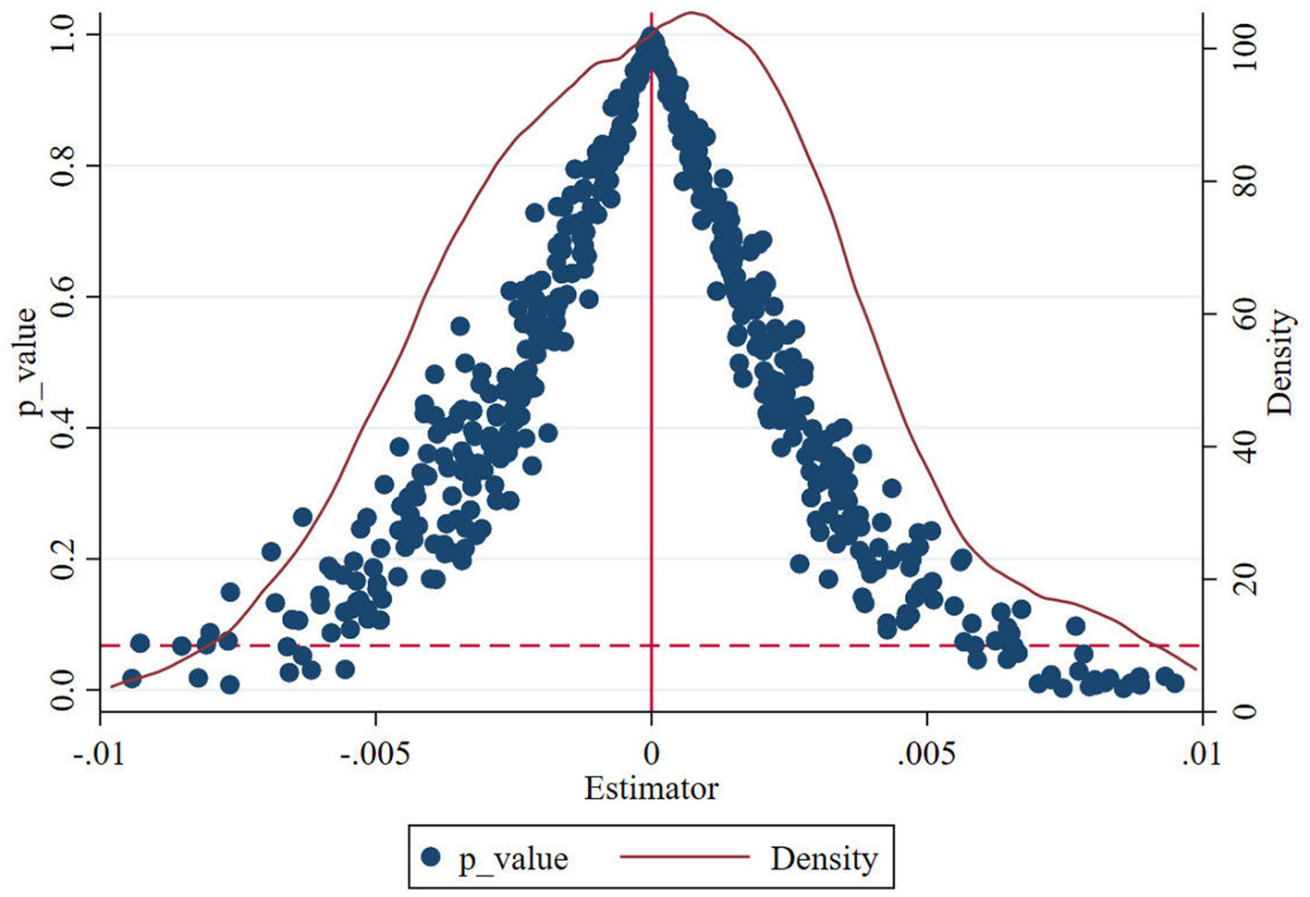
Results of placebo test.

Fourth, a placebo test is conducted to avoid the influence of other unobservable factors. Firstly, we randomly select the “experimental group” affected by the policy in the overall sample and then generates the policy time. Accordingly, the random experiment with two levels of pseudo experiment group and pseudo policy time is constructed, then we re-evaluate the previous estimates. Since the pseudo-policy time is randomly selected, the coefficient of the corresponding cross-product term *Pressure*×*Post* should theoretically be 0. We repeated the above operation 500 times in this study in case our estimation was accidental, and 500 coefficient estimates results are obtained. Figure 4 plots the kernel density and the corresponding *p*-value distribution of the estimates after 500 exercises. The curve denotes the kernel density distribution, the blue circle represents corresponding *p*-value, and the horizontal dotted line denotes the *p*-value which is 0.1. It is clear that the coefficient distribution corresponding to the pseudo-policy time concentrates around 0 and obeys the normal distribution, whereas the distribution of *p*-value indicates that the estimates of these coefficient all significantly reject the null hypothesis of coefficient of cross-product term. It illustrates that our aforementioned finding is not a coincidence of the experimental arrangement.

## 6 Heterogeneity analysis

### 6.1 Fiscal decentralization heterogeneity

Based on the previous theoretical analysis, this study further analyzes the impact of fiscal stress on local government public services provision under different fiscal decentralization. We split the samples into provinces with higher decentralization and lower decentralization to examine whether the results change. This study uses the ratio of local government general budget revenue to budget expenditure to measure the fiscal self-sufficiency rate (Lfa), and define provinces with more significant than the median of fiscal self-sufficiency rate as the higher fiscal decentralization group, and provinces with less significant than the median of fiscal self-sufficiency rate as the lower fiscal decentralization group. Table 6 reports the regression results. Columns (1) and (2) show the coefficient of lower fiscal decentralization group is 0.023, and it is significant at the 10% level, indicating that the impact of fiscal stress on public services provision is positive, while the coefficient of higher fiscal decentralization group is not significant. The results indicate that areas with lower fiscal decentralization can benefit from the central fiscal compensation mechanism (central government's fiscal transfer payments, tax attribution, etc.) to ensure the provision of public services when faced with fiscal stress. In areas with higher fiscal decentralization, due to the higher economic development and relatively abundant fiscal resources, there is less stress on public services provision when faced with fiscal stress. The empirical results support hypothesis 2.

**Table 6:** Heterogeneity test.

**Variable**	**Higher fiscal decentralization**	**Lower fiscal decentralization**	**High-intensity intergovernmental competition**	**Low-intensity intergovernmental competition**	**Higher fiscal transparency**	**Lower fiscal transparency**
	**(1)**	**(2)**	**(3)**	**(4)**	**(5)**	**(6)**
Stress	0.051	0.023^*^	0.039^***^	0.038^**^	0.059^**^	0.011^**^
	(0.034)	(0.013)	(0.012)	(0.017)	(0.027)	(0.005)
Control variables	Yes	Yes	Yes	Yes	Yes	Yes
Region FE	Yes	Yes	Yes	Yes	Yes	Yes
Year FE	Yes	Yes	Yes	Yes	Yes	Yes
Observations	150	150	150	150	150	150
*R*-squared	0.896	0.895	0.885	0.911	0.900	0.923

The values in parentheses are clustering with a robust standard error, ^*^, ^**^, and ^***^ represent the significance levels of 10%, 5%, and 1%, respectively.

### 6.2 Government competitive environment heterogeneity

This study further examines the differences in the impact of fiscal stress on public services provision under different intergovernmental competition environments. Since the more prefecture-level cities in the same province, the more intense inter-regional intergovernmental competition will be, so we use the number of prefecture-level cities in the province to measure the degree of competition among local governments (Gce). We defined provinces with more than the median of the degree of competition among local governments as the group with high-intensity intergovernmental competition, and provinces with less than this value will be considered the group with low-intensity intergovernmental competition. Columns (3) and (4) of Table 6 report the regression results for the high-intensity intergovernmental competition and the low-intensity intergovernmental competition provinces. The coefficients of both groups are positive and significant, but the coefficient of the group with high-intensity intergovernmental competition is 0.039 and significant at the 1% level, and the coefficient of the group with low-intensity intergovernmental competition is 0.038 and significant at the 5% level, which indicates that in an environment with high-intensity intergovernmental competition, fiscal stress has a more positive impact on the provision of local government public services. The more prefecture-level cities in the same province mean that the intergovernmental competition will be more intense, and the political promotion of local officials will be more difficult. And local officials are more inclined to maintain public services provision when subject to political championships and the central government's “protect people's livelihood” policy. The empirical results support the research hypothesis 3.

### 6.3 Fiscal transparency heterogeneity

This study further examines the differences in the impact of fiscal stress on public services provision under different fiscal transparency environments. We defined provinces with more than the median of the value of fiscal transparency as higher fiscal transparency areas, and province with less than the median of the value of fiscal transparency as lower fiscal transparency areas. The fiscal transparency data comes from the Chinese Government Transparency Index Report reported by Chinese Academy of Social Sciences. Since the data of 2016 did not report, we estimate data of 2016 with the sample data from 2013 to 2015 using the first-order autoregression analysis model, and the missing data in Nei Monggol, Guangxi, Ningxia, and Xinjiang in 2012 are processed by linear interpolation method. Columns (5) and (6) of Table 6 report the regression results. The coefficients of both groups are positive and significant at the 5% level, but the regression coefficient of group with higher fiscal transparency is higher than group with low fiscal transparency. The results indicate that areas with higher fiscal transparency have a more significant positive impact on the provision of public services than areas with lower fiscal transparency. On one hand, areas with high fiscal transparency can better manage and allocate fiscal resources under fiscal stress, by which promoting fiscal efficiency. On the other hand, high fiscal transparency will enhance the government's responsibility for fiscal decisions, reduce the possibility of fiscal waste and corruption, and enable more effective use of limited fiscal resources to provide public services in the face of fiscal stress. The empirical results support the hypothesis 4.

## 7 Discussion

This article regards China's VAT reform as a quasi-natural experiment. Based on the panel data of 30 provinces in China from 2012 to 2021, this study uses the generalized DID model to examine the impact of fiscal stress on the local governments provision of public services. After controlling the influence of year-fixed effects, region-fixed effects, and control variables, it is empirically concluded that Chinese local governments do not cut public services under fiscal stress but instead promote public services provision. In light of this finding, we asked the following discussion questions: What are the possible explanations for the results?

The possible reason for the results is the fiscal and political incentives created by Chinese-style decentralization. China's tax sharing reform in 1994 produced a more significant tendency of fiscal centralization. Although the central government grants local governments the autonomy in budget, the asymmetry of revenue decentralization and expenditure decentralization between the central and local governments leads to the weakening of local fiscal autonomy, and the local government's fiscal is also subject to performance appraisals by the central government, which may lead to the following results. On the one hand, due to the asymmetry of local government fiscal and administrative powers, local governments, especially those in low-decentralization areas, are more dependent on transfer payments from the central government. This system forms a soft budget constraint for local governments (Kornai, 1979), it also creates fiscal incentives for local governments to ensure the provision of public services when faced fiscal stress. On the other hand, the “three guarantees” has become the most basic functions of China's fiscal. The central fiscal has established a relatively complete “three guarantees” system and mechanism, and asks local fiscal to give priority to the “three guarantees.” The performance appraisal target may provide political incentives for local governments.

Based on the above analysis, it can be concluded that China's style of decentralization has actually established a “stress-based system.” This article posits that, in pursuit of meeting the central government's “three guarantees” performance targets and securing potential promotion opportunities, local government officials are more inclined to proactively respond to the central government's policy directives when facing fiscal constraints. They tend to prioritize safeguarding and enhancing public services as a key task in local fiscal expenditures. The results from the heterogeneity analysis also show that regions with high-intensity intergovernmental competition demonstrate a greater capacity to ensure the provision of public services when facing fiscal stress. However, as this study employs provincial-level data that cannot directly observe individual-level promotion incentives for officials, the aforementioned mechanism should be viewed as a possible theoretical logic rather than an empirically verified conclusion established in this research. Future studies incorporating micro-level evidence such as officials' tenure records and promotion data could provide more direct tests of this mechanism. Thus, China's style of decentralization reinforces the central government's wellbeing-oriented assessment targets and local governments' accountability for public service delivery. This dynamic represents both an inherent outcome of centralized decision-making by the central government when confronting significant fiscal stress and a strategic compliance choice by local governments acting as spending advocates.

## 8 Limitations and future research directions

This study also has several limitations that warrant investigation in future research.

Firstly, this study primarily treats provincial-level governments as the fundamental units and implementing agents for public service provision. It does not account for potential variations in expenditure behavior stemming from differing fiscal stress experienced by sub-provincial governments. In practice, sub-provincial governments, particularly grassroots entities such as cities and counties, also serve as fiscal decision-makers. For instance, county-level governments function as the primary implementing agents for education expenditures, accounts for approximately 80 % of the nation's expenditure on primary and secondary education. After the VAT reform, business tax, which was the largest tax source for local governments, was replaced by VAT, most of which is remitted to higher-level governments. However, the expenditure responsibilities of local governments at the county level have not been correspondingly reduced. As a result, county-level governments face more severe and complex fiscal constraints than higher-level governments. Further research could consider focusing on municipal and county-level governments, analyzing the response strategies and public service provision behaviors of governments at different levels under fiscal stress.

Secondly, this study examines the impact of macro factors such as fiscal decentralization, government competition, and fiscal transparency on the behavior of local governments in China. However, local governments typically face multi-objective governance issues. In addition to the aforementioned factors, micro-level factors such as promotion stress and the tenure of officials also influence decision-making under fiscal stress. Although the performance evaluation goals of local governments in China have gradually shifted from GDP-oriented targets to a focus on livelihood security, the promotion stress and limited tenure of local officials may still lead them to prioritize short-term projects with immediate results, rather than long-term livelihood spending. Consequently, short-term opportunistic behavior could significantly impact fiscal decisions during periods of fiscal stress. Therefore, short-term opportunistic behavior may affect local governments' decision-making under fiscal stress. Future research could explore how these micro-level factors influence the behavior of local governments.

Furthermore, this study mainly focuses on the overall level of public service supply by local governments, but does not further analyze whether fiscal stress leads to differences in the supply of different types of public services. Fiscal stress may affect the expenditure structure and priorities of local governments across different types of public services. For example, local governments may prioritize spending on basic public services such as primary education and healthcare, while reducing expenditures in other areas like culture and environmental protection. Therefore, future research could further explore the differences in the supply of various types of public services under fiscal stress.

## 9 Conclusions and policy implications

This article uses China's comprehensive implementation of VAT reform in 2016 as a quasi-natural experiment to test whether local governments promote the provision of public services under fiscal stress. Through empirical analysis, the main conclusions are as follows:

(1) Fiscal stress exerts a significant positive effect on local governments' provision of public services in China, serving as a catalyst for their service delivery. The results of the baseline regression still hold after a series of robustness tests. This result complements the existing research in related fields and makes up for the negligence of research on local government responses tor fiscal stress in developing countries. This empirical result provides a reference and basis for the design of China's budget system under fiscal stress and the budget execution of local governments.
(2) Heterogeneity analysis showed that there is difference of the impact of fiscal stress on local government public services provision in different areas. In areas with lower fiscal decentralization, high-intensity intergovernmental competition, higher fiscal transparency, fiscal stress is more conducive to promote public services provision. These empirical results indicate that the fiscal system and intergovernmental competition can affect the local government responses to fiscal stress. It makes up for the existing research on the effect of China's VAT reform, and the method used in this study can also be applied in emerging countries similar to China.

Based on the quasi-natural experimental study of China's VAT reform in 2016, we can see that China's fiscal system can effectively cope with the impact of fiscal stress and avoid the emergence of austerity cities. The research conclusions reflect that the fiscal centralization is conducive to strengthening the implementation of public service policy goals. The public nature of the Chinese government and the “top-down” stress system also enhance the fiscal incentives for public service expenditures, and the “three guarantees” policy goals can be achieved. At the same time, the fiscal stress brought about by the VAT reform has forced local governments to “tighten their belts,” reduce administrative expenses, ensure public service expenditures, and improve fiscal expenditure efficiency by optimizing expenditure structure. The Chinese style fiscal decentralization ensures that the central government can allocate financial resources uniformly when facing fiscal stress, guarantees financial support for local government public service expenditures, and improves the execution of central policies. This is also the reason why local governments in China have been able to ensure and provide high-quality local public services in the past 3 years in response to COVID-19 pandemic.

Based on the above research conclusions, the following policy implications are further proposed.

First, establish a graded fiscal stress response mechanism and a dynamic allocation of responsibilities between the central and local governments. Create a fiscal stress response system to clarify the dynamic responsibilities of central and local governments in financing, allocation, and execution of public services under different levels of fiscal stress. Develop an automatic stress threshold recognition system and a central-local responsibility transition protocol framework. When a decline in fiscal self-sufficiency triggers a stress signal, adjust the central-local responsibilities accordingly to ensure that in times of crisis, the central government can swiftly take over standardized service provision, while in normal times, it can release more space for local innovation.

Second, optimize the expenditure structure and improve the efficiency of fiscal spending. Continuously optimize the fiscal expenditure structure, directing funds with long-term insufficient benefits toward key livelihood security areas. Focus on the performance of public service spending by applying scientific methods to track the direction and efficiency of funds to ensure that fiscal resources in key areas are effectively utilized.

Third, design a public service-oriented performance competition system for local governments. Restructure the incentive mechanism for intergovernmental competition by incorporating core public service indicators such as the quality of basic education, accessibility to basic healthcare, and social security coverage into the performance evaluation framework. Establish a reward and penalty system based on relative performance evaluation, providing budget flexibility rewards for high-performing regions, and linking these rewards to fiscal incentives, transfer payments, and bond issuance quotas, while also incorporating public satisfaction evaluations.

Fourth, implement institutional reforms for full-process fiscal transparency. Legislate the establishment of disclosure standards for fiscal information throughout the entire cycle, from budget preparation to policy performance evaluation, with a focus on strengthening the tracking and disclosure of financial flows in the public service sector. Develop a fiscal resilience monitoring indicator system to regularly assess the risk-bearing capacity of local governments, providing a basis for risk warnings and expenditure adjustments.

Finally, it must be noted that this article uses China as a case study to examine the behavior of local governments under fiscal stress, which represents a unique example of the rich and diverse practice of fiscal decentralization worldwide. China's experience has certain reference value for countries with similar “political centralization combined with economic decentralization” fiscal management systems, as it helps unify the actions of central and local governments, strengthens local governments' implementation of central policies, and ensures the achievement of central political objectives. However, due to the differences in fiscal management systems across countries, as well as variations in the political and economic goals of central governments and the incentives and constraints imposed on local governments, the experiences and policy recommendations derived from this article have certain limitations.

## Data Availability

The original contributions presented in the study are included in the article/Supplementary material, further inquiries can be directed to the corresponding author.

## Footnotes

^1^The fiscal self-sufficiency ratio is defined as the ratio of local general public budget revenue to local general public budget expenditure (Gu et al., 2022).

^2^The 30 provinces include Beijing, Tianjin, Hebei, Liaoning, Shanghai, Jiangsu, Zhejiang, Fujian, Shandong, Guangdong, Hainan, Heilongjiang, Jilin, Shanxi, Anhui, Jiangxi, Henan, Hubei, Hunan, Inner Mongolia, Guangxi, Chongqing, Sichuan, Guizhou, Yunnan, Shaanxi, Gansu, Qinghai, Ningxia, and Xinjiang.
